# NikA/TcsC Histidine Kinase Is Involved in Conidiation, Hyphal Morphology, and Responses to Osmotic Stress and Antifungal Chemicals in *Aspergillus fumigatus*

**DOI:** 10.1371/journal.pone.0080881

**Published:** 2013-12-02

**Authors:** Daisuke Hagiwara, Azusa Takahashi-Nakaguchi, Takahito Toyotome, Akira Yoshimi, Keietsu Abe, Katsuhiko Kamei, Tohru Gonoi, Susumu Kawamoto

**Affiliations:** 1 Medical Mycology Research Center, Chiba University, Chiba, Japan; 2 New Industry Creation Hatchery Center, Tohoku University, Sendai, Japan; Universidade de Sao Paulo, Brazil

## Abstract

The fungal high osmolarity glycerol (HOG) pathway is composed of a two-component system (TCS) and Hog1-type mitogen-activated protein kinase (MAPK) cascade. A group III (Nik1-type) histidine kinase plays a major role in the HOG pathway of several filamentous fungi. In this study, we characterized a group III histidine kinase, NikA/TcsC, in the life-threatening pathogenic fungus, *Aspergillus fumigatus*. A deletion mutant of *nikA* showed low conidia production, abnormal hyphae, marked sensitivity to high osmolarity stresses, and resistance to cell wall perturbing reagents such as congo red and calcofluor white, as well as to fungicides such as fludioxonil, iprodione, and pyrrolnitrin. None of these phenotypes were observed in mutants of the SskA response regulator and SakA MAPK, which were thought to be downstream components of NikA. In contrast, in response to fludioxonil treatment, NikA was implicated in the phosphorylation of SakA MAPK and the transcriptional upregulation of *catA*, *dprA*, and *dprB*, which are regulated under the control of SakA. We then tested the idea that not only NikA, but also the other 13 histidine kinases play certain roles in the regulation of the HOG pathway. Interestingly, the expression of *fos1*, *phkA*, *phkB*, *fhk5*, and *fhk6* increased by osmotic shock or fludioxonil treatment in a SakA-dependent manner. However, deletion mutants of the histidine kinases showed no significant defects in growth under the tested conditions. Collectively, although the signal transduction network related to NikA seems complicated, NikA plays a crucial role in several aspects of *A. fumigatus* physiology and, to a certain extent, modulates the HOG pathway.

## Introduction

*Aspergillus fumigatus* is a major causative pathogen of invasive aspergillosis (IA) worldwide. This fungus infects immunocompromised patients, and IA is known for its relatively high mortality [1]. Despite recent progress in diagnostic and therapeutic modalities, IA is still one of the most life-threatening infectious diseases. One reason for this is that the antifungal options to combat this pathogen are very limited. Thus, the identification of molecular targets for new antifungal medications is an urgent issue.

The two-component system (TCS) is a signal transduction system that is conserved in a wide range of organisms from bacteria to higher plants, but not in mammals [2], [3]. In general, it senses environmental stimuli by sensor histidine kinases (HKs), transmits signals, and leads to appropriate cellular responses with response regulators (RRs). The molecular basis of signaling is the His-to-Asp phosphorelay system, in which a phosphorus group directly and reversibly transfers between conserved histidine and asparagine residues in HK and RR domains, respectively. The fungal TCS has hybrid-histidine kinases (hHKs), which have both HK and RR domains, and a histidine-containing phosphotransfer protein (HPt) as an intermediate factor. As a consequence, the fungal TCS functions as a multistep phosphorelay composed of hHKs, HPt, and RRs (His-Asp-His-Asp) [4].

Molecular analysis of the fungal TCS has been intensively performed in *Saccharomyces cerevisiae*, in which a single hHK, Sln1p, a single HPt, Ypd1p, and two response regulators, Ssk1p and Skn7p, have been shown to constitute TCS signaling. The TCS is directly linked to the stress-activated mitogen-activated protein kinase (MAPK; SAPK) cascade and regulates its activation in response to osmotic conditions in the extracellular environment, resulting in a high osmolarity glycerol (HOG) pathway [5], [6]. To date, the link to the MAPK cascade has also been observed in several filamentous fungi including plant and human pathogens [7]. In model filamentous fungi such as *Neurospora crassa* and *Aspergillus nidulans*, the TCS plays a role in a variety of physiological cellular functions including conidiation, sexual development, oxidative stress response, osmotic adaptation, and sensitivity to fungicides [8]–[12]. In some plant pathogenic fungi, the TCS plays a crucial role in pathogenicity, and is supposedly the target of the phenylpyrrole and dicarboximide classes of fungicides that are widely used to protect crops in the agricultural industry [13]–[15]. The accumulation of these findings supports the idea that the TCS is a promising molecular target for new antifungal therapies against human pathogens, including *A. fumigatus* [16].

In this decade, genome data have become available for several fungi, which allowed us to quickly search for components of the TCS and examine the diversity and universality of the fungal TCS. Catlett et al. (2003) revealed that the number of hHKs in filamentous fungi is generally greater than 10, which presented a sharp contrast to the numbers observed in yeast such as *S. cerevisiae* (1 hHK), *Schizosaccharomyces pombe* (3 hHKs), and *Candida albicans* (3 hHKs) [17]. In *A. fumigatus*, 13 HKs have previously been identified, and three (Fos1, TcsB, and TcsC) of them have been investigated. A deletion mutant of the *fos1* gene showed a moderate resistance to fungicides and attenuated virulence [18], [19]. The *tcsB* deletion mutant showed a slight sensitivity to sodium dodecyl sulfate (SDS) and growth inhibition under high temperature conditions [20], [21]. TcsC, a group III histidine kinase, was recently characterized by McCormick et al. as described below [22]. Characterization of the other HKs would be helpful to improve the understanding of the TCS signaling circuitry in *A. fumigatus*.

Among the different types of HKs, a large amount of attention has been paid to group III (Nik1-type) HKs. This gene (*os-1*/*nik1*) was initially identified in *N. crassa* as an osmotic stress-sensitive mutant allele, and later it was identified as a dicarboximide-resistant mutant allele [12], [23]. This HK possesses a characteristic motif in its N-terminal region, consisting of four to six repeats of the HAMP (histidine kinases, adenylyl cyclases, methyl-accepting chemotaxis proteins, and phosphatases) domain. Although the functions of the motif were obscure, a null mutation and deletion of the gene resulted in resistance to the dicarboximide and phenylpyrrole fungicides in all fungi that possess this type of HK in its genome [10], [12], [24]–[28]. Intriguingly, although *S. cerevisiae* has no Nik1-type HK, heterologous expression of Nik1-type HKs from other species made *S. cerevisiae* responsive to these fungicides [29]–[33]. These findings illustrated that Nik1-type HKs play a crucial role in the fungicide action and that the mode of action is convertible across some fungi. Furthermore, a recent striking finding for this type of HK is its involvement in dimorphic switching in dimorphic pathogens including *Penicillium marneffei*, *Histoplasma capsulatum*, and *Blastomyces dermatitidis* [28], [34]. However, the detailed molecular mechanism has yet to be elucidated.

Recently, the characterization of TcsC, a Nik1-type HK of *A. fumigatus*, has been reported by McCormick et al. [22]. TcsC is required for hyper-osmotic stress adaptation and sensitivity to certain fungicides such as fludioxonil, as well as phosphorylation of the SakA MAPK in response to these stimuli. Despite these phenotypes *in vitro*, TcsC seemed to be dispensable for virulence. In this study, we characterized the Nik1-type HK of *A. fumigatus* (NikA/TcsC) with regard not only to its role in osmotic stress and fungicide responses, development, and morphology, but also with regard to its role in the signaling pathway associated with the SskA response regulator and SakA MAPK, which are downstream components of the HOG pathway. Furthermore, the involvement of the other HKs in the HOG pathway was investigated. Based on our findings, we discussed the molecular mechanism of the TCS circuitry and the stress response mechanism of the HOG pathway in *A. fumigatus.*

## Results

### NikA is Required for Normal Conidiation

**Figure 1:**
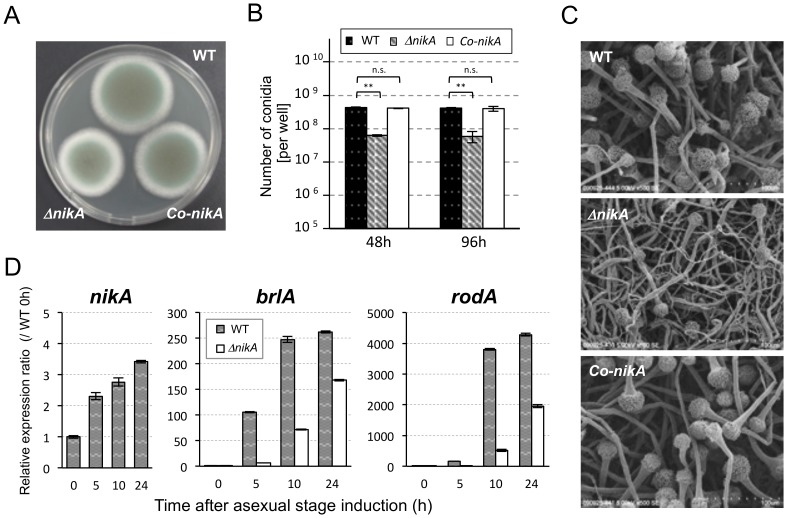
Deletion of the *nikA* gene results in a conidiation defect. (**A**) Conidial suspensions of the parental strain, Δ*nikA*, and the *nikA*-complemented strain (*Co-nikA*) were point-inoculated on 0.1% yeast extract containing glucose minimal medium (YGMM) agar plates and incubated for 68 h at 37°C. (**B**) The number of conidia in a 34 mm diameter well of a 6-well plate after 48 h and 96 h of incubation at 37°C was counted (See Materials and Methods). Error bars represent the standard deviations based on three independent replicates. **P<0.001 versus WT strain at any time point. *P*-values were calculated by the Student’s *t*-test. (n.s.: not significant) (**C**) The colony surface was observed by scanning electron microscopy (SEM). The growth conditions were the same as those in (B): 48 h incubation at 37°C in 6-well plates. (**D**) The expression of *nikA*, *brlA*, and *rodA* during the asexual development stage was determined by real-time reverse transcriptase polymerase chain reaction (RT-PCR). To synchronize asexual development initiation, mycelia that were cultured in liquid YGMM for 18 h were harvested and transferred onto YGMM plates (the time point was set as 0 h of the asexual stage). Relative expression ratios were calculated relative to WT at 0 h. Error bars represent the standard deviations based on three independent replicates.

To investigate the physiological role of the NikA/TcsC HK, we constructed a deletion mutant of the *nikA* gene. Afs35 (*akuA::loxP*) was used as a host strain (WT), and the *nikA* gene was replaced with the hygromycin-resistant marker [35]. Three independent transformants (Δ*nikA*) were obtained through a standard transformation procedure, and the deletion of the *nikA* gene was confirmed at both the genomic and expression levels (data not shown). First, colony growth and conidia production were examined in the *nikA* deletion mutant (named Δ*nikA* hereafter). On a 0.1% yeast extract-containing glucose minimal medium (YGMM) plate, the colony diameter of Δ*nikA* was 82.5±0.8% of the WT (Figure 1A, Table S1). Also, Δ*nikA* produced fewer conidia than WT after 48 h and 96 h of incubation (Figure 1B). The germination and viability rates of Δ*nikA* conidia were indistinguishable from those of WT conidia (data not shown). Scanning electron microscopy (SEM) observations showed that although the conidiophore structure was normal in Δ*nikA*, the number of conidiophores was reduced (Figure 1C). To confirm these defects, we constructed *nikA*-complemented strains (named *Co-nikA*). The fitness and number of conidia were restored in *Co-nikA* (Figure 1A to C), indicating that these phenotypes resulted from the absence of the *nikA* gene.

To confirm the observed differences in conidia production, we examined the expression level of the *nikA*, *brlA*, and *rodA* genes during asexual development. *brlA* encodes a master regulator of conidiation, whose expression is increased in early asexual development [36]. *rodA* encodes a hydrophobin, which is a major conidia coating protein in *A. fumigatus* [37]. We found that in the WT strain the expression level of the *nikA* gene was increased as asexual development proceeded (Figure 1D). Consistent with the reduction in conidia, the expression levels of *brlA* and *rodA* were lower in Δ*nikA* than in WT (Figure 1D). These results suggest that NikA plays an important role in asexual development and is required for a full level of conidia production.

### NikA Affects the Resistance to Cell Wall–Perturbing Agents

**Figure 2:**
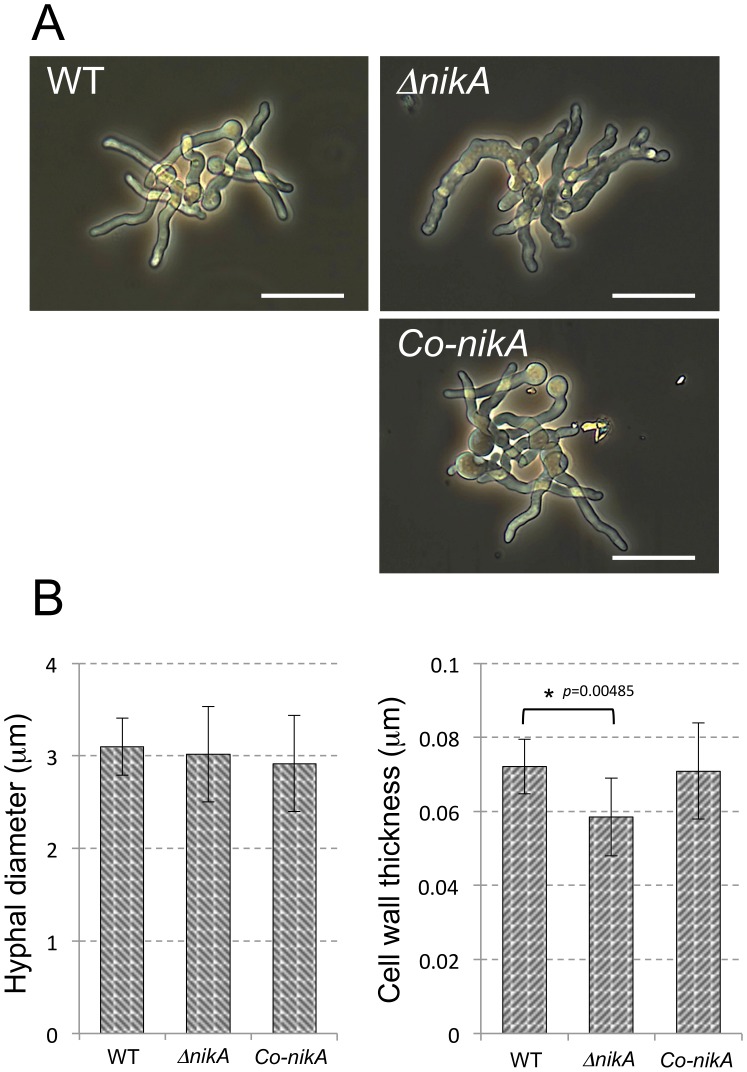
Defects of the cell wall structure in Δ*nikA*. (**A**) Conidia were incubated for 9 h in YGMM. The germlings were observed under optical microscopy. The bar represents 20 µm. (**B**) The cell wall thickness and the diameter of hyphae were examined from the hyphal cross-section. In each cross-section, the cell wall thickness was examined from at least 10 different positions. The largest and smallest measurements were eliminated, and the rest of values were averaged. The column indicates the mean value of the hyphal diameter (left) and cell wall thickness (right) from 10 different cross-sections (n = 10). Error bars represent the standard deviations. *P*-values versus WT were calculated by the Student’s *t*-test.

**Figure 3:**
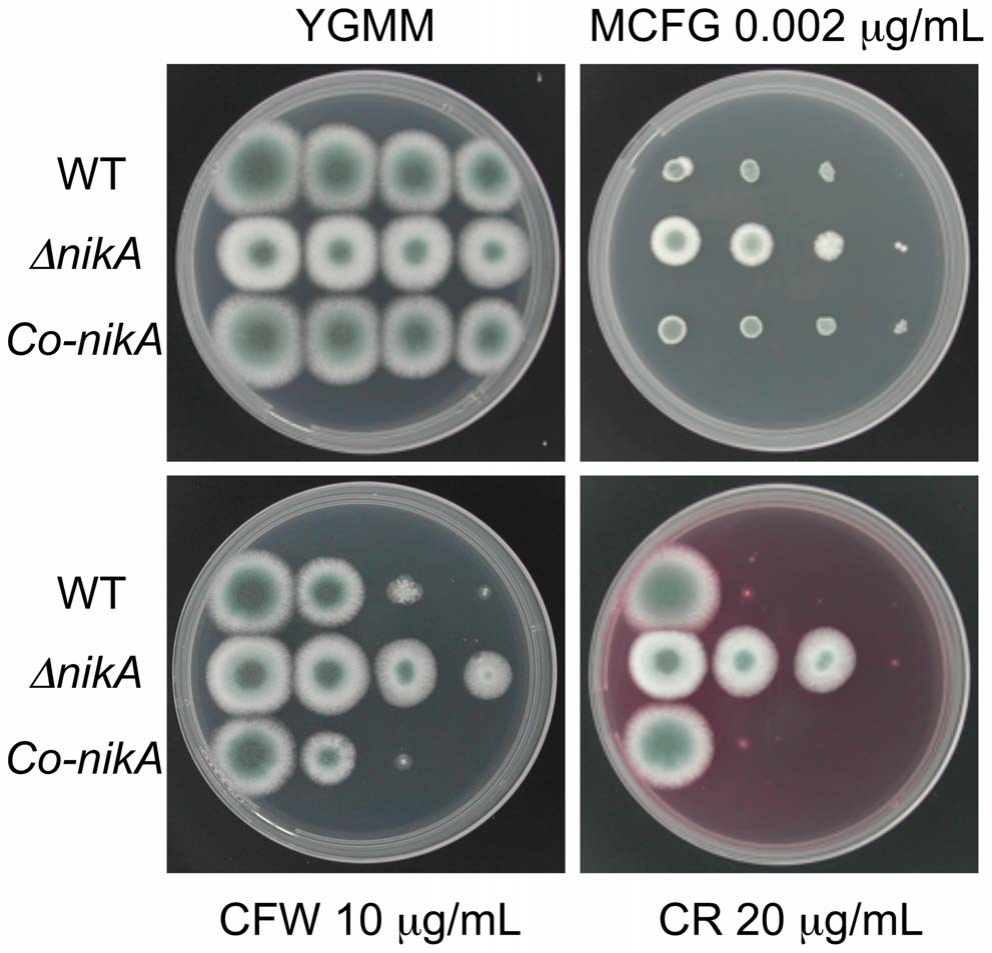
Growth of Δ*nikA* on plates containing cell wall-perturbing reagents. Series of diluted conidia suspensions (left to right: 10^4^, 10^3^, 10^2^, and 10 conidia) of WT, Δ*nikA*, and *Co-nikA* were spotted onto YGMM plates with or without micafungin (MCFG), calcofluor white (CFW), or congo red (CR) at the indicated concentration. These plates were incubated at 37°C for 44 h and photographed.

Because the radial growth of Δ*nikA* was slightly disrupted, we compared the morphological trait of growing hyphae in Δ*nikA* and WT. Microscopy observations revealed that the hyphal shape of Δ*nikA* was obviously different from that of WT and *Co-nikA* under liquid culture conditions (Figure 2A). In fact, Δ*nikA* showed rough and aberrant hyphae. To gain more insight into the cell wall structure, we observed the hyphae by transmission electron microscopy (TEM). Hyphal diameter and cell wall thickness were determined from the images of hyphal cross-sections, which showed that the cell wall of Δ*nikA* was slightly thinner than that of WT and *Co-nikA* cells (Figure 2B). These abnormalities of Δ*nikA* hyphae led us to examine the sensitivity to cell wall-perturbing agents such as congo red (CR), calcofluor white (CFW), and micafungin (MCFG). Δ*nikA* showed significant resistance to CR and CFW compared with WT, whereas it only showed slight resistance to MCFG (Figure 3). Taken together these findings suggest that the cell wall structure of Δ*nikA* is different from that of WT.

We next examined the cell wall carbohydrate composition in WT and Δ*nikA* strains. The cell wall of both strains was fractionated into alkali-soluble (AS1 and AS2) and alkali-insoluble (AI) fractions. Alpha-1,3-glucan is largely fractionated in AS2, while beta-1,3-glucan and chitin are largely fractionated in AI. Galactomannan is fractionated in both AS2 and AI. The amounts of glucose or glucosamine in the AS2 and AI fractions were largely similar between Δ*nikA* and WT, indicating that the amounts of alpha-1,3-glucan, beta-1,3-glucan and chitin were indistinguishable (Table S2). Notably, there was a difference in the galactosamine contents between Δ*nikA* and WT. Polygalactosamine, alpha-1,3-glucan, and galactomannan are thought to compose an amorphous cement in cell wall structure [38]. Thus, the reduction in galactosamine contents in Δ*nikA* may result in a modification of the cell wall structure that explains the resistances to cell wall-perturbing agents. However, the detailed function of galactosamine in the cell wall structure remains elusive. In any event, these results suggest that although the major cell wall carbohydrate composition of Δ*nikA* was comparable to that of WT, NikA affected hyphal morphology and resistance to cell wall stressors.

### NikA is Required for Osmotic Stress Adaptation and Sensitivity to Fungicides

**Figure 4:**
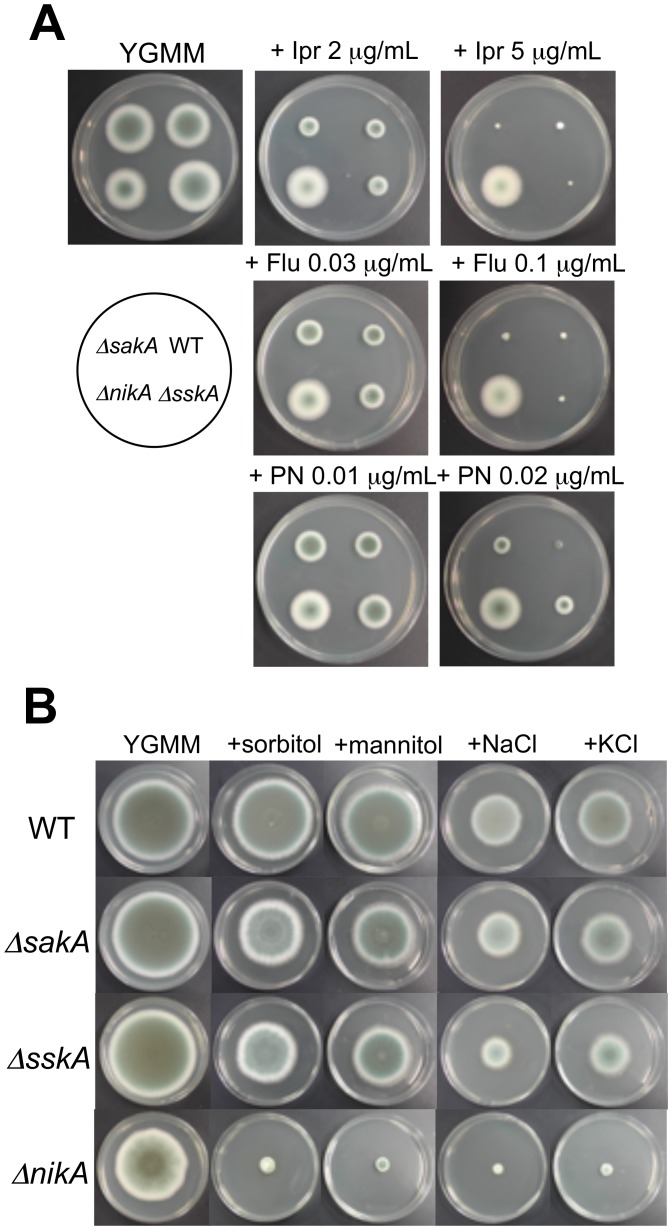
Growth of *sakA*, *sskA*, and *nikA* deletion mutants under high osmotic or fungicide stress conditions. (**A**) Growth on plates containing fungicides. Conidia of Δ*sakA*, Δ*sskA*, Δ*nikA*, and WT were inoculated onto YGMM containing iprodione (Ipr), fludioxonil (Flu), or pyrrolnitrin (PN) at the indicated concentrations and were incubated at 37°C for 48 h. (**B**) Growth on plates containing high osmotic stress. Conidia of Δ*sakA*, Δ*sskA*, Δ*nikA*, and WT were inoculated onto YGMM containing 1.2 M sorbitol, 1.2 M mannitol, 1 M NaCl, or 1 M KCl and were incubated at 37°C for 96 h.

As reported in several studies, the Nik1-type HK is involved in fungicide action and osmotic stress adaptation in most fungi [24]–[31]. Thus, we sought to determine whether Δ*nikA* shows resistance to fungicides and sensitivity to osmotic stress. Prior to the evaluation of fungicide susceptibility of Δ*nikA*, the growth inhibitory concentration (IC) of the WT was determined. Fludioxonil, a phenylpyrrole fungicide, and iprodione, a dicarboximide fungicide, inhibited the radial growth on YGMM plates at concentrations above 0.01 µg/mL and 0.5 µg/mL, respectively, while pyrrolnitrin had a superior inhibiting activity (Figure S1). Based on these tests, the growth of Δ*nikA* was examined on plates containing appropriate concentrations of fungicides (roughly estimated concentrations of IC50 and IC90 of the WT strain), revealing that Δ*nikA* had substantial resistance to the fungicides (Figure 4A). We next investigated the growth of Δ*nikA* on YGMM plates containing high concentrations of sorbitol, mannitol, NaCl, or KCl as high osmolarity stress conditions. Δ*nikA* showed impaired growth on the osmotic stress plates tested here (Figure 4B). Notably, resistance to fungicides and sensitivity to osmotic stresse of Δ*nikA* were also observed in liquid culture (data not shown), and these growth phenotypes were restored in *Co-nikA* (Figure S2). These results indicate that NikA plays an important role in the sensitivity to fungicides and osmotic stress adaptation in *A. fumigatus* as it does in other filamentous fungi.

### SskA and SakA MAPK are not Required for Conidiation, Hyphal Morphology, and Sensitivity to the Fungicides

We next asked how NikA plays its role in the cellular functions and responses described above. Several lines of evidence have suggested that the NikA HK directly interacts with the HPt protein, YpdA, and modulates the SakA MAPK cascade via SskA RR. Thus, we decided to assess the involvement of the downstream components in conidia production, hyphal morphology, sensitivity to cell wall-perturbing reagents and fungicides, and osmotic stress resistance. We constructed mutant strains of the *sakA* and *sskA* genes (named Δ*sakA* and Δ*sskA*, respectively). Δ*sakA* and Δ*sskA* showed slightly faster radial growth (Δ*sakA*: 106.6±0.9% and Δ*sskA*: 104.0±1.2%) and comparable colony morphology and conidia production on YGMM with those of the WT (Figure 4B, Table S1, and data not shown). Light microscopy observations revealed that Δ*sakA* and Δ*sskA* displayed normal hyphae (data not shown). We also found that Δ*sakA* and Δ*sskA* were not as resistant to CR, CFW, and MCFG as Δ*nikA* was (Figure S3). With regard to fungicide resistance, Δ*sakA* and Δ*sskA* were as sensitive to fludioxonil and iprodione as the WT (Figure 4A and Table S1). Notably, Δ*sakA* and Δ*sskA* showed a slight resistance to pyrrolnitrin, suggesting that the SskA-SakA MAPK pathway is involved, to some extent, in the action of pyrrolnitrin (Figure 4A and Table S1). When grown on YGMM plates containing high osmotic stresses Δ*sakA* and Δ*sskA* showed significant, but less than that observed in Δ*nikA*, growth retardation (Figure 4B and Table S1). This result suggests that SskA and SakA play a partial role in the adaptation to high hyperosmotic environments, while NikA is crucial for it. Taken together, SskA and SakA were dispensable for normal conidiation, hyphal morphology, and resistance to cell wall-perturbing reagents and fungicides, suggesting that NikA plays a role in these cellular functions via a signaling pathway that is distinct from the SskA-SakA MAPK pathway.

### SakA MAPK is Phosphorylated by Osmotic Shock and Fungicide Treatment via SskA

**Figure 5:**
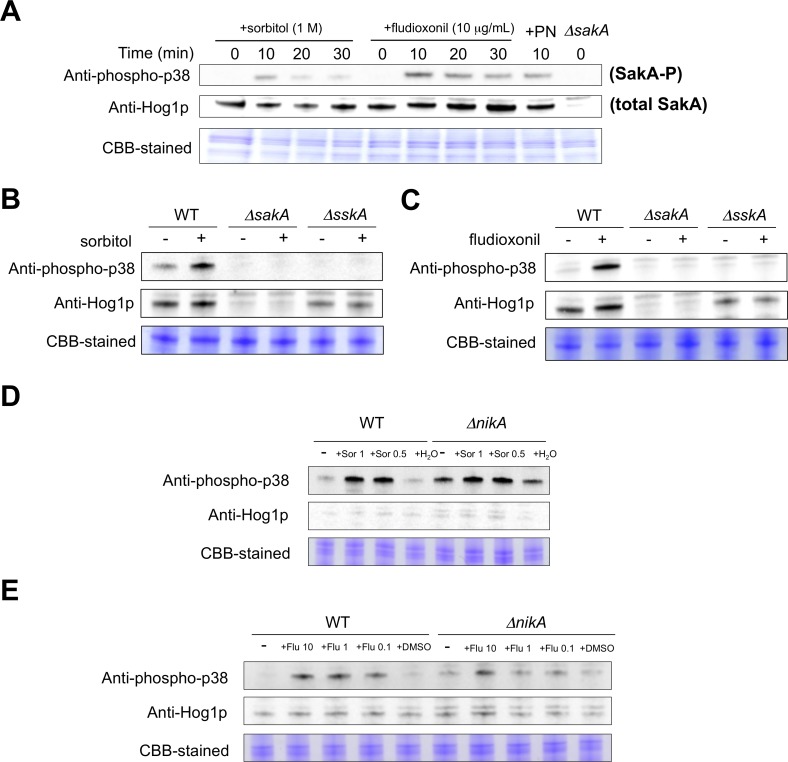
Immunoblot analysis for phosphorylation of the SakA MAPK in response to osmotic or fungicide stress. (**A**) The WT strain was grown for 18 h at 37°C. Then, sorbitol (1 M final concentration), fludioxonil (10 µg/mL final concentration), or pyrrolnitrin (1 µg/mL) was added. The mycelium was harvested at the indicated times, and total proteins were extracted. Anti-phospho-p38 was used to detect the phosphorylation of SakA, and anti-Hog1p was used to detect the total protein of SakA. A Coomassie Brilliant Blue (CBB)-stained gel is shown as a loading control. (**B** and **C**) WT, Δ*sakA*, and Δ*sskA* were grown with or without sorbitol (1 M final concentration) (B) or fludioxonil (10 µg/mL final concentration) (C). CBB-stained gels are shown as loading controls. (**D** and **E**) WT and Δ*nikA* were grown with or without sorbitol (0.5 and 1 M final concentrations) (D) or fludioxonil (0.1, 1, and 10 µg/mL final concentrations) (E). CBB-stained gels are shown as loading controls. The same volume of water (D) or DMSO (E) was added to the culture instead of sorbitol or fludioxonil as a control.

We next determined whether NikA is involved in the activation of the SakA MAPK cascade in response to environmental stimuli. It should be noted that besides osmotic stress and fungicides, Δ*nikA*, as well as Δ*sakA* and Δ*sskA*, displayed no significant growth differences when exposed to oxidative stress (hydrogen peroxide and menadione), antifungal chemicals (miconazole, fluconazole, and amphotericin B), and high temperature (42°C) compared with WT (data not shown). Thus, we focused on responses to osmotic stress and fungicides to define the signal transduction from the TCS to the SakA MAPK cascade in *A. fumigatus*. To monitor the SakA MAPK activation, we examined the phosphorylation level of SakA protein by immunoblot analysis with the commercial antibodies, anti-phospho-p38 MAPK (Thr180/Tyr182) and anti-Hog1 (y-215) (See Material and Methods). The phosphorylation of SakA was detected 10 min after the addition of sorbitol (1 M final concentration), and the levels decreased at 20 and 30 min (Figure 5A). When treated with fludioxonil or pyrrolnitrin, SakA was also phosphorylated (Figure 5A). These results suggest that SakA is rapidly activated after osmotic or fungicide treatments, and then the phosphorylated form gradually decreases.

Next, we investigated whether NikA and SskA are involved in the phosphorylation of the SakA MAPK. In Δ*sskA*, no phosphorylated SakA was detected with osmotic shock and fludioxonil treatment, while the total SakA protein was obviously present (Figure 5B and C). This indicates that SskA is indispensable for the phosphorylation of the SakA MAPK. In contrast, an increased level of SakA phosphorylation was detected in Δ*nikA* without stimulus, suggesting that the absence of NikA led to SakA phosphorylation (Figure 5D and E). Although the phosphorylation level was high in the absence of stimuli in Δ*nikA* cells, the addition of 1 M or 0.5 M sorbitol, or 10 µg/mL fludioxonil further increased the phosphorylation levels (Figure 5D and E). Interestingly, when treated with 1 or 0.1 µg/mL fludioxonil, there was no apparent increases in the phosphorylated form of SakA in Δ*nikA*, while phosphorylated SakA accumulated in WT. These results suggest that NikA plays a major role in SakA phosphorylation in response to fludioxonil treatment, but not in response to osmotic shock.

### Osmotic Shock and Fungicide Induce Transcription of *catA*, *dprA*, and *dprB* through the HOG Pathway

To gain more insight into the role of NikA in mediating the SakA MAPK cascade, we examined the expression of genes that are possibly regulated under the control of the SakA MAPK. In our previous study on *A. nidulans*, *catA*, which encodes a conidia-specific catalase A, was regulated in a SakA/HogA MAPK cascade-dependent manner (unpublished data). Hence, we used the corresponding *A. fumigatus catA* to monitor the activation of the SakA MAPK cascade. In addition, the recently reported SakA-regulated genes *dprA* and *dprB*, both encoding a dehydrin-like protein, were also used for the expression analysis [39]. First, we investigated whether the expression of these genes was regulated in response to osmotic shock and fludioxonil treatment. After the addition of the stimuli, *catA*, *dprA*, and *dprB* were upregulated and the peak of expression was at 15–30 min (Figure S4).

**Figure 6:**
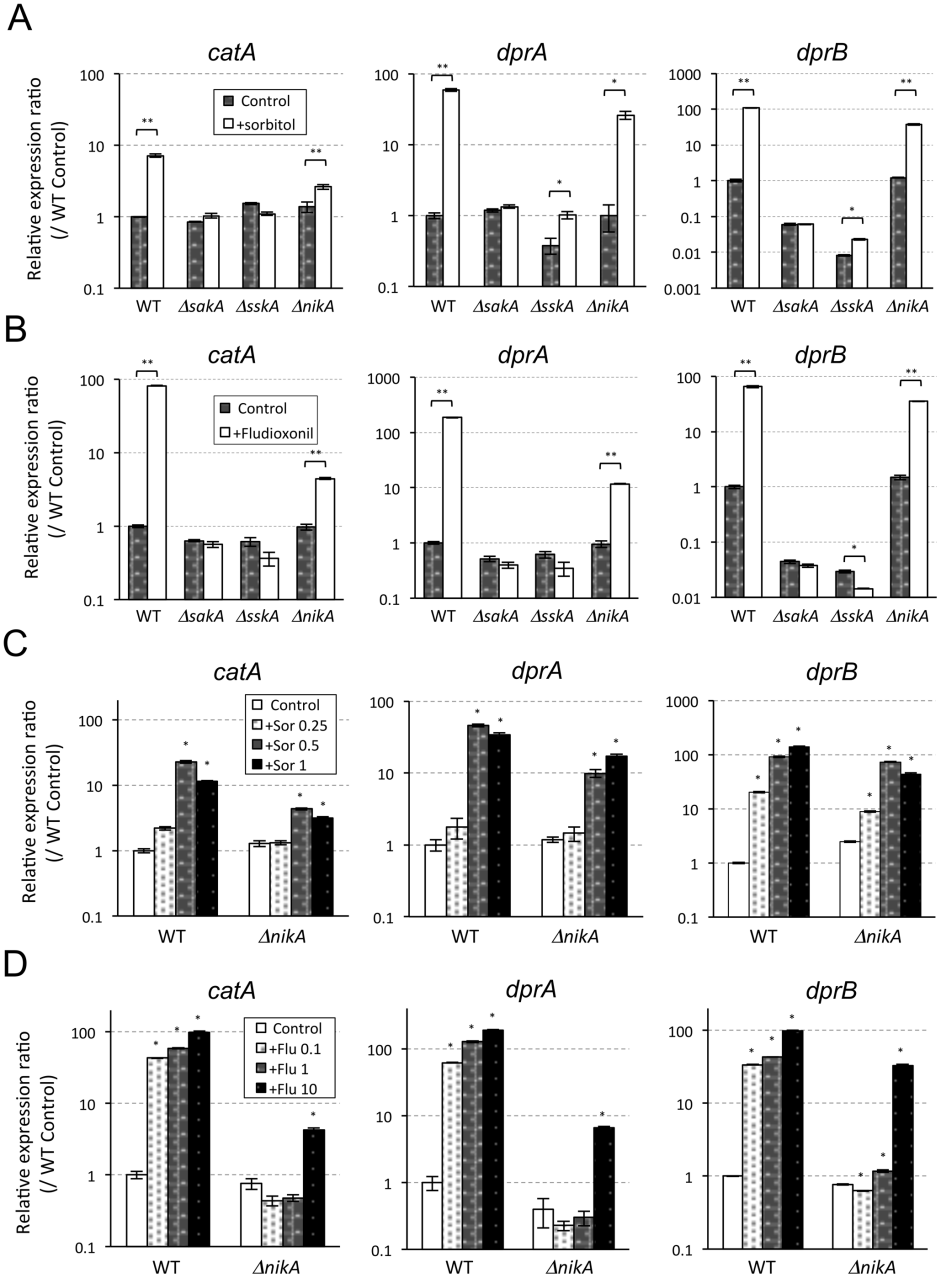
Transcriptional responses of *catA*, *dprA*, and *dprB* to osmotick shock and fungicide. (**A** and **B**) WT, Δ*sakA*, Δ*sskA*, and Δ*nikA* were grown in liquid YGMM for 18 h at 37°C. Then, sorbitol (A) or fludioxonil (B) was added (1 M or 10 µg/mL final concentrations, respectively). As a control, an equivalent volume of water or DMSO was added to the cultures (indicated as Control). After 15 min, the mycelium was harvested, and the RNA was extracted. cDNAs was synthesized and used for real-time RT-PCR analysis. Relative expression ratios were calculated relative to the WT control. Error bars represent the standard deviations based on three replicates. *P*-values were calculated by the Student’s *t*-test: *P<0.005; **P<0.001, compared to the Control in any strain. (**C** and **D**) WT and Δ*nikA* were grown in liquid YGMM for 18 h at 37°C. Then, different concentrations of sorbitol (0.25, 0.5, and 1 M final concentrations) (C) or fludioxonil (0.1, 1, and 10 µg/mL final concentrations) (D) were added. After 15 min, the mycelium was harvested, and the RNA was extracted. cDNAs was synthesized and used for real-time RT-PCR analysis. Relative expression ratios were calculated relative to WT. Error bars represent the standard deviations based on three replicates. *statistically significant difference relative to the Control in any strain (*P*<0.05). P-values were calculated by student’s t-test followed by Bonferroni method.

Next, the upregulation of these genes (15 min after adding each stimulus) was investigated in Δ*nikA*, Δ*sakA*, and Δ*sskA*. In Δ*sakA* and Δ*sskA*, there was virtually no increase in the expression of any of the genes in response to osmotic shock (1 M sorbitol) or fludioxonil (10 µg/mL) treatment (Figure 6A and B). These results indicated that the upregulation of *catA*, *dprA*, and *dprB* was exclusively dependent on SskA and the SakA MAPK. In comparison, the expression of these genes significantly, but not completely, increased in response to osmotic shock and fludioxonil in Δ*nikA*, suggesting that the transcriptional responses of *catA*, *dprA*, and *dprB* are not fully dependent on NikA (Figure 6A and B). To further confirm this partial response, we examined the expression levels in WT and Δ*nikA* with various concentrations of sorbitol (0.25, 0.5, and 1 M) and fludioxonil (0.1, 1, and 10 µg/mL). When treated with 0.5 M sorbitol, the expression was significantly upregulated in WT as well as in Δ*nikA* (Figure 6C). In response to the serial concentrations of fludioxonil, Δ*nikA* was upregulated only with the 10 µg/mL treatment while upregulation was observed in WT even with the 0.1 µg/mL treatment. These results suggest that NikA is responsible for the transcriptional responses of *catA*, *dprA*, and *dprB* to the lower concentrations (0.1 and 1 µg/mL) of fludioxonil through the SskA-SakA pathway.

### Expression of some Histidine Kinase Genes is Regulated through the SakA MAPK

**Figure 7:**
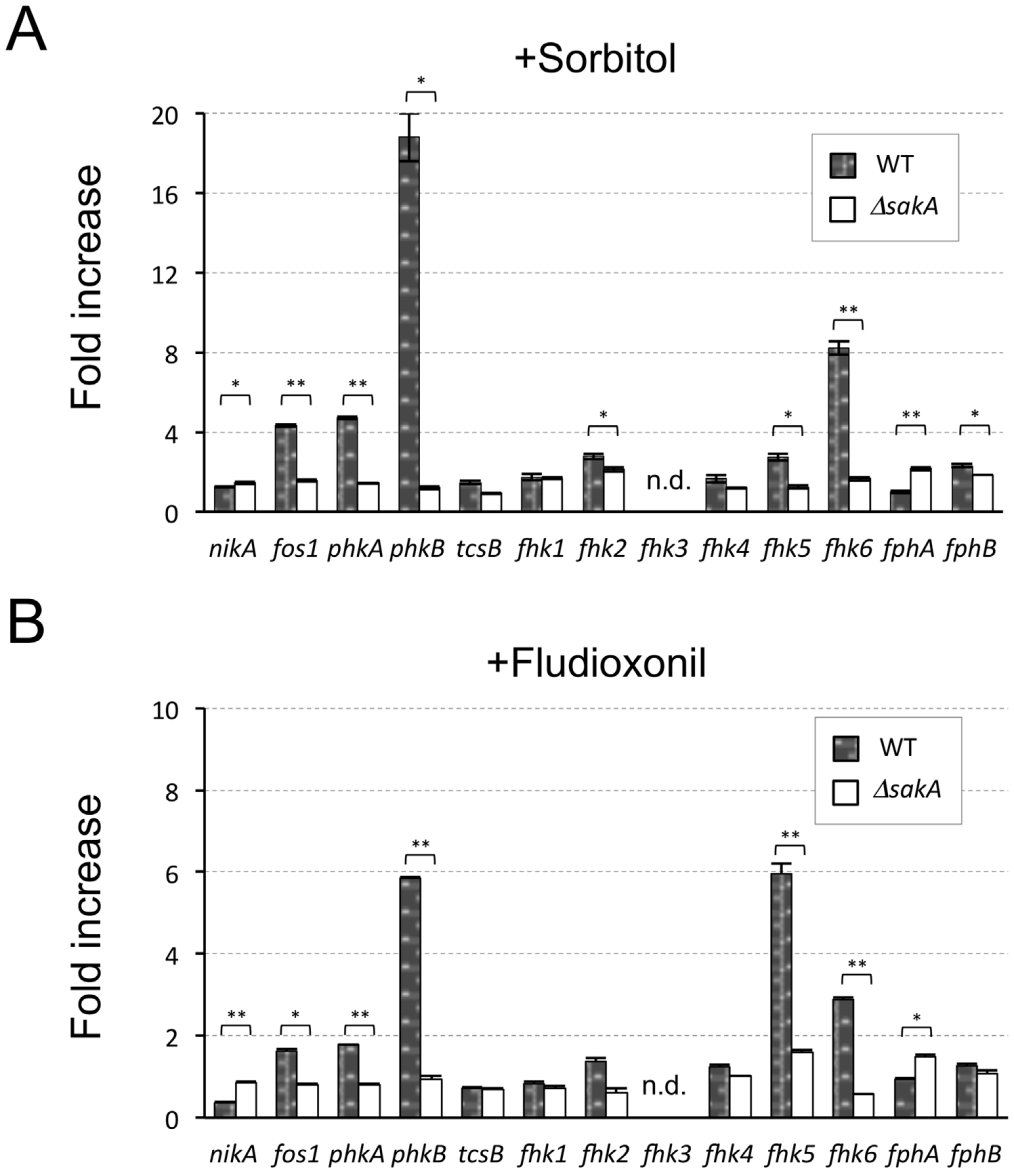
Transcriptional responses of *A. fumigatus* histidine kinase (HK) genes to osmotic shock and fungicide. (**A** and **B**) WT and Δ*sakA* were grown in liquid YGMM for 18 h at 37°C. Then, sorbitol (A) or fludioxonil (B) was added (1 M and 10 µg/mL final concentrations, respectively). After 15 min, the mycelium was harvested, and the RNA was extracted. cDNAs was synthesized and used for real-time RT-PCR analysis. The relative expression ratios were compared between samples with and without osmotic shock or fludioxonil treatment; the fold increases of each HK gene in WT and Δ*sakA* were determined. Error bars represent the standard deviations based on three replicates. *P<0.005; **P<0.001 versus WT. *P*-values were calculated by the Student’s *t*-test. (n.d.: not determined).

The finding that the transcriptional responses to osmotic stress and fungicides were fully dependent on SskA and partly dependent on NikA led us to ask whether the other HKs play a compensative role for mediating the SakA MAPK cascade in Δ*nikA* cells. To examine the involvement of the other HKs in the HOG pathway, the expression level of all 13 HK genes in response to the osmotic stress and fludioxonil treatment was determined in WT and Δ*sakA*. *fhk3* expression was not detected by our real-time reverse transcriptase-polymerase chain reaction (RT-PCR) system in several trials using different primer sets. In response to osmotic shock, the expression of *fos1*, *phkA*, *phkB*, and *fhk6* was markedly upregulated (>4-fold), and this upregulation was dependent on the SakA MAPK (Figure 7A). Likewise, *phkB* and *fhk5* were upregulated in a SakA MAPK-dependent manner by fludioxonil treatment (Figure 7B). These results suggest the possibility that Fos1, PhkA, PhkB, Fhk5, and Fhk6 play a certain role in the modulation of the SskA-SakA MAPK pathway in response to these stimuli.

To investigate whether Fos1, PhkA, PhkB, Fhk5, and Fhk6 are implicated in osmotic stress adaptation and fungicide action, we constructed the deletion mutants Δ*fos1*, Δ*phkA*, Δ*phkB*, Δ*fhk5*, and Δ*fhk6*. Because Δ*phkA* could not be constructed in the Afs35 background, we created Δ*phkA* in the Af293 background and compared it to Af293. The colony growth of the mutants was investigated on a plate containing hyperosmotic stress or fungicides. Although Δ*phkB* colonies showed slight growth retardation on YGMM plates, these mutants showed a virtually wild-type growth rate on YGMM plates with or without hyperosmotic stress or fungicides (Figure S5). These results suggest that none of these HKs solely plays a role in the osmotic stress adaptation and fungicide action.

To reveal whether these HKs play a compensative role for NikA in modulating the SakA MAPK cascade, double deletion mutants should be constructed. Prior to this, we investigated the expression profiles of these HKs in Δ*nikA* cells that were treated with osmotic shock or fludioxonil. None of the HKs showed higher expression in Δ*nikA* than in WT (data not shown). This result led us to assume that none of these HKs were activated, at least at the expression level, in the cells lacking NikA. Therefore, we did not investigate the double deletion mutants in this study. Collectively, these results suggest that whereas NikA plays an important role in modulating the SakA MAPK cascade, none of the other HKs affect the HOG pathway functions.

## Discussion

In this study, we found that the absence of NikA resulted in pleiotropic phenotypes such as growth retardation, reduction of conidia, aberrant hyphae, tolerance to cell wall-perturbing reagents and fungicides, and marked sensitivity to high osmolarity stress. However, NikA is likely to function independently of SskA and SakA in these phenotypes except for osmotic adaptation, which raised the question of what alternative component functions downstream of NikA. Because NikA is a component of the His-Asp phosphorelay circuitry in the TCS, NikA is thought to regulate RRs via the YpdA HPt in a phosphorelay-dependent manner. Given that SskA is not involved in the phenotypes, a plausible candidate is AfSkn7, the other type of RR in *A. fumigatus*. In *A. nidulans*, the double gene deletion of SskA and SrrA, which is an ortholog of AfSkn7, resulted in a Δ*nikA*-level tolerance to the fungicides and a marked sensitivity to osmotic stress [10]. A similar result was also reported in *Cochliobolus heterostrophus*, suggesting that SskA and SrrA/Skn7 are redundantly or cooperatively involved in the adaptations to fungicides and osmotic environment downstream of NikA [13]. Importantly, *A. nidulans* single deletion mutants of *sskA* or *srrA* showed only slight resistance to fungicides and a moderate sensitivity to the osmotic stress [9], [10]. Another research group characterized AfSkn7, where AfSkn7 was implicated in the oxidative stress response but not in morphology and pathogenicity in a murine model of aspergillosis [40]. In addition, the *afskn7* deletion mutant constructed by our group showed no significant resistance to cell wall-perturbing reagents and fungicides (unpublished data). Thus, to better understand how NikA regulates conidiation, morphology, and responses to chemicals, the generation and investigation of a double deletion mutant of *sskA* and *afskn7* is necessary. This will be the subject of our future study.

As in other fungi, the *A. fumigatus* SakA MAPK was transiently phosphorylated by osmotic shock and fungicide treatment, which was fully dependent on SskA. This clearly indicates that under the conditions tested here the SakA MAPK cascade is solely regulated by the TCS. Unexpectedly, we found that SakA was phosphorylated in response to osmotic shock in Δ*nikA* cells, suggesting that another mechanism modulates the SakA phosphorylation instead of NikA, possibly via SskA. On the one hand, NikA seemed to be indispensable for the accumulation of phosphorylated SakA in response to low concentrations of fludioxonil treatment (up to 1 µg/mL), whereas it was dispensable when treated with 10 µg/mL fludioxonil. Judged by the roughly estimated growth ICs (0.03 and 0.1 µg/mL as IC50 and IC90, respectively), 10 µg/mL of fludioxonil seems high and may cause a leaky effect at a specific interaction between the chemical and the target protein. In *C. heterostrophus* and *N. crassa*, the accumulation of phosphorylated Hog1-type MAPK was observed in the cells lacking the Nik1-type HK in the presence of high concentrations of fungicides or high osmolar reagents [41]. These results could support the idea that another HK or mechanism contributes to the phosphorylation of SakA via SskA in some filamentous fungi. Notably, in contrast to our results, in a previous study by McCormick et al. (2012), TcsC/NikA has been shown to be required for the phosphorylation of SakA in response to fludioxonil treatment (10 µg/mL) and osmotic shock (1.2 M sorbitol) in *A. fumigatus* [22]. We think that this discrepancy is derived from the differences in media (AMM vs. YGMM), cultivation time (9 h vs. 18 h), and treatment time (2 min vs. 15 min for fludioxonil, and 20 min vs. 15 min for osmotic shock). Considering these results, the regulation of the SakA MAPK cascade by NikA may be growth stage-specific.

We also found that the phosphorylation level of the SakA MAPK in Δ*nikA* was moderately high in the absence of stimuli. This increased phosphorylation level is reminiscent of a role of the *S. cerevisiae* Sln1p in modulating the Hog1 MAPK phosphorylation, where a defect in the *sln1* gene causes abnormal activation (hyper-phosphorylation) of the Hog1 MAPK and leads to growth defects [6]. In fact, Δ*nikA* showed a slight defect in radial growth on YGMM plates and increased the phosphorylation of SakA. These findings could support the idea that similarly to Sln1p NikA is a negative regulator of the SakA MAPK, whereas it plays a positive role at least in response to fludioxonil treatment. Here, it should be noted that the expression level of *catA*, *dprA*, and *dprB* in Δ*nikA* in the absence of stimuli was comparable to that in WT, suggesting that the moderate accumulation of phosphorylated SakA dose not necessarily lead to the high expression of the genes. There may be unknown regulatory mechanisms, for example a negative feedback system, concerning this. In any event, judged by the results of SakA phosphorylation and transcriptional responses to the stimuli, the phosphorylated form of SakA appears to be an active form. Also, we showed a straightforward signaling from SskA to the SakA MAPK in the *A. fumigatus* HOG pathway both at the transcriptional and post-translational levels.

In hyperosmotic stress conditions, the Δ*nikA*, Δ*sskA*, and Δ*sakA* strains showed growth sensitivity at distinct levels. As has been well studied in *S. cerevisiae* [42], [43], the HOG pathway plays an important role in osmotic adaptation by regulating glycerol accumulation; thus, it is possible that glycerol accumulation occurred in response to hyperosmotic stress conditions in *A. fumigatus*, and the HOG pathway was responsible for this. In addition, DprB is a dehydrin-like protein responsible for growth under hyperosmotic conditions [39]. This seems to be one of the reasons for the growth retardation of Δ*sskA* and Δ*sakA*, in which not only the transcriptional response to osmotic shock but also the basal level of *dprB* expression were impaired. On the other hand, moderate levels of the transcriptional response and basal expression of *dprB* were observed in Δ*nikA*. These findings presented the possibility that the considerable growth impairment of Δ*nikA* under osmotic stress conditions resulted from not only a shut-off of the SskA-SakA-DprB function but also from a defect in another component. As stated above, the Skn7-type RR and SskA additively function in the osmotic adaptation of several fungi [9], [10], [13]. Thus, AfSkn7 may be a downstream component of NikA; together, they play an important role in osmotic adaptation.

In general, the fungal TCS is a target of fungicides such as fludioxonil, iprodione, and pyrrolnitrin, and the Nik1-type HK is supposedly the molecular target [10], [12], [15], [25], [31], [44]–[48]. We revealed that the *A. fumigatus* NikA was indispensable for fungicide activity (growth inhibitory effects). This result is consistent with the results of studies on other fungi. As stated above, relatively high concentrations of fludioxonil may have a leaky effect on HOG pathway activation. With regard to this, we hypothesized that non-specific interactions between fludioxonil and the other HKs occurred during the 10 µg/mL fludioxonil treatment. To examine this, we tried to clarify any implication of other HKs in this effect, but no obvious interplay was observed. These results allowed us to suggest another possibility: fludioxonil interacts directly with YpdA protein or the NikA-YpdA complex to inhibit its function. The reason for this assumption is twofold. First, as in most fungi including *S. cerevisiae*, *N. crassa*, *Cryptococcus neoformans*, and *A. nidulans* [6], [48]–[50], *afypdA* is likely to be an essential gene (unpublished data). Second, when the expression of *afypdA* is downregulated, growth is inhibited and the SakA MAPK pathway is activated (unpublished data). These phenomena are reminiscent of how cells react to fludioxonil treatment. The clarification of this hypothesis is under investigation in our group.

A detailed molecular mechanism of the mode of action of fungicides such as fludioxonil is one of the long-sought topics concerning the biological control of plant pathogenic fungi [51], [52]. The fungicides used here have a broad spectrum, and they affect animal pathogenic fungi such as *Candida*, *Cryptococcus*, *Aspergillus*, and *Fusarium* species [7]. Hence, this type of chemical seems to be a promising compound for antifungal drugs for life-threatening deep mycoses [53]. The elucidation of the molecular target, the signaling pathway, and the mode of action in each pathogen will aid the development of newly designed drugs with a broad spectrum.

In general, the TCS of filamentous fungi is composed of more than 10 HKs, two to four RRs, and one HPt factor [7], [17]. In *A. fumigatus*, only a few HKs have been characterized so far [18]–[22]. To gain more comprehensive insight into the *A. fumigatus* TCS, we determined the expression profiles of all HKs. Interestingly, some HKs were upregulated in response to osmotic shock or fludioxonil treatment, and they were dependent on the SakA MAPK. We assumed that these HKs play certain roles in the signaling of the SakA MAPK cascade. From investigations of the deletion mutants, however, we had no clear evidence for this assumption. Notably, Fos1 has been reported to be involved in pathogenicity, sensitivity to fungicides, and cell wall construction [18], [19]. Our *fos1* deletion mutant, however, showed no role in sensitivity to fungicide and cell wall stress reagents. We think that this discrepancy of phenotypes is derived from the different background of the strain. In addition, Δ*phkA*, Δ*phkB*, Δ*fhk5*, and Δ*fhk6* showed no visible phenotypes (colony size, surface color, and conidiation ability), including susceptibility to hydrogen peroxide, fungicides, and cell wall stressors, when compared with WT (data not shown). To elucidate the physiological role of these HKs, further functional analyses are necessary. Recently, Ji et al. (2012) have reported that TcsB plays an important role in growth in a high-temperature environment and likely modulates the SakA MAPK phosphorylation state in *A. fumigatus* [21]. Taken together with findings in this study, it appears that a robust and complex signal transduction system for adaptation to extracellular environments exists in *A. fumigatus*.

In conclusion, we found that NikA plays an important role in conidiation, morphology, and stress responses, and that the SakA MAPK cascade is regulated through SskA (TCS) in response to osmotic shock and fungicide treatment. We also characterized the other HKs including Fos1, PhkA, PhkB, Fhk5, and Fhk6. There were no interactions between these HKs and NikA or SakA, at least under the conditions tested here. Although NikA seems to be dispensable in *A. fumigatus* pathogenicity, molecular insights provided in this study may open possibilities in the development of new antifungal chemicals focusing on the TCS signaling of medically relevant fungal pathogens.

## Materials and Methods

### Strains and Growth Media

*A. fumigatus* strain Afs35 (FGSC A1159) (*akuA::loxP*) was used to generate the following gene deletion strains: Δ*nikA*, Δ*sakA*, Δ*sskA*, Δ*fos1*, Δ*phkB*, Δ*fhk5*, and Δ*fhk6* [35]. *A. fumigatus* strain Af293 was used to generate Δ*phkA*. These genes (*nikA*, Afu2g03560; *sakA*, Afu1g12940; *sskA*, Afu5g08390; *fos1*, Afu6g10240; *phkA*, Afu3g12550; *phkB*, Afu3g12530; *fhk5*, Afu4g00660; and *fhk6*, Afu4g00320) were replaced with the hygromycin resistance marker (*hph* or HygB^r^), sequences of which were derived from plasmids pSH75 (a generous gift from Dr. Takashi Tsuge, Nagoya University) or pCB1004, respectively [54]. An ectopic *nikA*-complemented strain (*Co-nikA*) was generated by transformation with a fragment containing *nikA* on pPTR I (Takara BIO, Ohtsu, Japan) (see below). Fungal strains used in this study are listed in Table 1. All strains were routinely cultivated in 0.1% yeast extract containing glucose minimal medium (YGMM) at 37°C. To collect conidia of each strain, potato dextrose agar (PDA) was used. For plates containing osmotic stress, 1.2 M sorbitol, 1.2 M mannitol, 1 M NaCl, or 1 M KCl was added. In liquid culture, 20 mL of 3 M sorbitol or 2.4 M NaCl was added to 40 mL of YGMM (final concentration, 1 M or 0.8 M, respectively). For antifungal chemicals, a 1000-fold concentrated stock solution was prepared and added to media in appropriate concentrations. Fludioxonil, iprodione, and MCFG were a generous gift from Kumiai-Chem. Co. Pyrrolnitrin (from *Pseudomonas cepacia*), CFW (fluorescent brightener 28), and CR were commercially obtained (Sigma-Aldrich Co., St. Louis, MO, USA).

**Table 1:** Fungal strains used in this study.

Strain	Description	Reference
Afs35 (FGSC A1159)	Wild type (*akuA::loxP*)	From FGSC
Af293	Wild type	From FGSC
Δ*nikA*	*nikA::HygB^r^*	This study
*Co-nikA*	*nikA::HygB^r^+nikA*	This study
Δ*sskA*	*sskA::HygB^r^*	This study
Δ*sakA*	*sakA::hph*	This study
Δ*fos1*	*fos1::HygB^r^*	This study
Δ*phkA*	*phkA::HygB^r^*	This study
Δ*phkB*	*phkB::HygB^r^*	This study
Δ*fhk5*	*fhk5::HygB^r^*	This study
Δ*fhk6*	*fhk6::HygB^r^*	This study

### Construction of the Gene Deletion and Reconstituted Strains

To construct the *A. fumigatus* deletion and the reconstituted strains, plasmids for each purpose were generated. DNA manipulation was performed according to standard laboratory procedures for recombinant DNA. To amplify DNA fragments from the genome and prepare gene replacement cassettes using one-step fusion PCR, Platinum Taq DNA Polymerase High Fidelity (Invitrogen, Carlsbad, CA, USA) or PrimeSTAR HS (Takara BIO) were used. Plasmids for transformation were constructed by the GeneArt system (Invitrogen) or one-step fusion PCR [55]. Primers used in this study are listed in Table S3.

For the generation of pSH75-sakA, 5′- and 3′-flanking regions of the *sakA* gene were obtained using the primers sakA-U(Bgl)-FW and sakA-U(Bgl)-RV (for the 5′-region) and sakA-D(Xho)-FW and sakA-D(Xho)-RV (for the 3′-region). The fragments were digested using the restriction enzymes *Bgl*II and *Xho*I and were ligated into pSH75 carrying the *hph* cassette. The resulting plasmid, pSH75-sakA, was used for transformation to construct the *sakA* deletion strain.

For the generation of plasmid pUC119-nikA, 5′- and 3′-flanking regions of the *nikA* gene were obtained using the primers nikA-U(Pst)-FW and nikA-U-RV-(HygBr) (for the 5′-region) and nikA-D-FW(HygBr) and nikA-D(Pst)-RV (for the 3′-region). The fragment of the HygB^r^ cassette was obtained from the pCB1004 plasmid using the primers HygBr-FW and HygBr-RV. HygB^r^ flanked by the *nikA*-flanking regions was obtained by one-step fusion PCR. The fragment was digested with the restriction enzyme *Pst*I and was ligated into *Pst*I-linearized pUC119. The resulting plasmid, pUC119-nikA, was used for transformation to construct the *nikA* deletion strain.

For the generation of plasmid pUC119-sskA, 5′- and 3′- flanking regions of the *sskA* gene were obtained using the primers sskA-U-F(BamHI) and sskA-U-RV(HygBr) (for the 5′-region) and sskA-D-FW(HygBr) and sskA-D-R(EcoRI) (for the 3′-region). HygB^r^ flanked by the *sskA*-flanking regions was obtained by one-step fusion PCR. The fragment was digested with the restriction enzymes *Bam*HI and *Eco*RI and was ligated into pUC119 that was linearized with *Bam*HI and *Eco*RI. The resulting plasmid, pUC119-sskA, was used for transformation to construct the *sskA* deletion strain.

For the generation of plasmid pUC119-fos1, 5′- and 3′-flanking regions of the *fos1* gene were obtained using the primers fos1A-U(Pst) and fos1-U-R(HygBr) (for the 5′-region) and fos1-D-F(HygBr) and fos1-D(Pst) (for the 3′-region). HygB^r^ flanked by the *fos1*-flanking regions was obtained by one-step fusion PCR. The fragment was digested with the restriction enzyme *Pst*I and was ligated into pUC119 that was linearized with *Pst*I. The resulting plasmid, pUC119-fos1, was used for transformation to construct the *fos1* deletion strain.

For the generation of plasmids pUC119-phkA, pUC119-phkB, pUC119-fhk5, and pUC119-fhk6, 5′- and 3′-flanking regions of each gene were obtained using the primers phkA-U2-F(pUC119B) and phkA-U2-R(HygBr) (for the 5′-region of *phkA*), phkA-D-F(HygBr) and phkA-R(pUC119E) (for the 3′-region of *phkA*), phkB-F(pUC119B) and phkB-U-R(HygBr) (for the 5′-region of *phkB*), phkB-D-F(HygBr) and phkB-R(pUC119E) (for the 3′-region of *phkB*), fhk5-F(pUC119B) and fhk5-U-R(HygBr) (for the 5′-region of *fhk5*), and fhk5-D-F(HygBr) and fhk5-R(pUC119E) (for the 3′-region of *fhk5*), fhk6-F2(pUC119B) and fhk6-U-R(HygBr) (for the 5′-region of *fhk6*), and fhk6-D-F(HygBr) and fhk6-R2(pUC119E) (for the 3′-region of *fhk6*). These flanking regions and the HygB^r^ fragment were fused into pUC119 using the GeneArt system, resulting in pUC119-phkA, pUC119-phkB, pUC119-fhk5, and pUC119-fhk6, which were used for transformation to construct Δ*phkA*, Δ*phkB*, Δ*fhk5*, and Δ*fhk6*, respectively.

For the generation of plasmid pPTRI-nikA+, the *nikA* fragments containing 5′- and 3′-flanking regions were obtained using the primers nikA-N-F(pPTR-P) and nikA-N-R (for the fragment containing the 5′-region and N-terminus of *nikA*), and nikA-C-F and nikA-C-R(pPTR-K) (for the fragment containing the 3′-region and C-terminus of *nikA*). These two fragments were fused into pPTRI using the GeneArt system, resulting in pPTRI-nikA+, which was used for transformation of Δ*nikA* to construct the ectopically complemented strain *Co*-*nikA*.

*A. fumigatus* transformation was performed according to conventional methods for protoplast-polyethylene glycol (PEG) transformation for *Aspergillus*. Briefly, a mycelium that was cultured for one night was harvested from YGMM liquid medium and was treated with Lysing Enzymes (Sigma-Aldrich Co.) and Yatalase (Takara BIO) for more than 3 h to obtain a sufficient amount of protoplasts. The DNA was coincubated with the protoplasts, and osmotic treatment with PEG4000 led to the incorporation of the DNA. The protoplast was mixed into agar medium containing 1.2 M sorbitol and appropriate antibiotics for selection.

Homologous recombination and gene replacement were confirmed by PCR of the genomic DNA, and the absence of the mRNA of the target gene was verified using real-time RT-PCR analysis. We obtained at least three independent transformants for each gene deletion mutant, and the phenotypes of the multiple strains were clarified.

### Conidia Preparation and Culture Conditions

Conidia of each strain were stored in a deep freezer (–80°C) to prevent unexpected mutations. To prepare fresh conidia suspensions, the stored conidia were inoculated on a PDA slant and incubated at 37°C for four to five days. Conidia were harvested with phosphate-buffered saline (PBS) containing 0.1% Tween 20 and kept at 4°C. Throughout this study, conidia were freshly harvested every two weeks. The number of conidia was counted using a hemocytometer (Watson, Kobe, Japan).

For the synchronized induction of asexual development, conidia (10^5^ conidia/mL) were cultivated in liquid YGMM for 18 h, and mycelia were harvested using Miracloth (Merck, Frankfurt, Germany), washed with distilled water, and transferred onto YGMM plates. These plates were incubated at 37°C (this time point was set as 0 h), and the mycelia were harvested at the appropriate times.

### Colony Diameter Measurement

About 10^4^ conidia of each strain were point-inoculated on YGMM plates. After incubation for 72 h at 37°C, the diameter of the colony was measured (n = 3), and the mean values were calculated. The growth ratio under stressed conditions was calculated by comparing the radial diameter on the YGMM plate with and without antifungal chemicals or osmotic stress reagents. To judge sensitivity or resistance to the stresses, the calculated ratio of the diameter of each strain was compared with that of WT.

### Calculation of Conidia Numbers

Before the agar solidified, conidia were mixed into YGMM agar medium (final concentration, 10^4^ conidia/mL). The medium containing conidia was poured into a 6-well plate (3 mL per well), solidified, and then incubated at 37°C. After 48 h or 96 h of incubation, the agar from each well including mycelia and produced conidia was vortexed in 10 mL of PBS containing 0.1% Tween 20 for 1 min. The number of conidia in the suspensions was counted using a hemocytometer.

### RNA and cDNA Preparation

The mycelia were harvested and frozen in liquid nitrogen, and total RNA was isolated using the FastRNA Pro Red Kit (MP Biomedicals, Santa Ana, CA, USA). The possible contaminating DNA was digested with Deoxyribonuclease for Heat Stop (Wako Pure Chemical Industries, Osaka, Japan). To obtain cDNA pools from the total RNA, reverse transcription was performed using the High Capacity cDNA Reverse Transcription Kit (Life Technologies Corporation, Carlsbad, CA, USA).

### Quantitative Real-Time RT-PCR

Real-time RT-PCR was performed using the 7300 system (Life Technologies Corporation) with SYBR Green detection according to the manufacturer’s instructions. For reaction mixture preparation, the THUNDERBIRD SYBR qPCR Mix was used (TOYOBO, Osaka, Japan). The primers listed in Table S3 were used to quantify the gene expression of interest. Primer specificity was verified by disassociation analysis. A cDNA sample that was obtained from reverse transcription reactions using 1 µg of total RNA was applied to each reaction mixture. The *actin* gene was used as the normalization reference (internal control) for target gene expression ratios, and WT without stress or at 0 h was set as the calibrator in each experiment. Relative expression ratios were calculated by first calculating the threshold cycle changes in sample and calibrator as 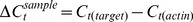 and 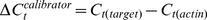, followed by calculating the expression rates in both sample and calibrator as 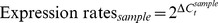 and 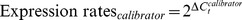, and finally, 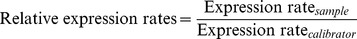. Each sample was tested in triplicate. For biological replicates, each test was repeated at least twice on different days.

### Immunoblot Analysis

Conidia (final concentration, 10^5^/mL) were inoculated into liquid YGMM and cultured for 18 h prior to addition of sorbitol or fungicides. Mycelia were collected with Miracloth, frozen in liquid nitrogen, ground to a powder, and immediately suspended in protein extraction buffer containing protease inhibitors [50 mM Tris-HCl, pH 7.5, 1% sodium deoxycholate, 1% Triton X-100, 0.1% SDS, 50 mM NaF, 5 mM sodium pyrophosphate, 0.1 mM sodium vanadate, and protease inhibitor cocktail (Wako)]. The suspension was immediately boiled for 10 min with an appropriate sample buffer for SDS-polyacrylamide gel electrophoresis (PAGE), and cell debris was then removed by centrifugation for 10 min at 14,000 rpm. The protein concentration of the supernatant was determined using a Pierce BCA Protein Assay Kit–Reducing Agent Compatible (PIERCE, Rockford, IL, USA). Total protein was loaded onto a NuPAGE Novex Bis-Tris 4–12% gel (Invitrogen) and blotted using the iBlot gel transfer system (Invitrogen). To detect total SakA and phospho-SakA proteins, a rabbit polyclonal IgG antibody against Hog1 y-215 (sc9079, Santa Cruz Biotechnology, Santa Cruz, CA, USA) and a rabbit polyclonal IgG antibody against dually phosphorylated p38 MAPK (#4631, Cell Signaling Technology, Beverly, MA, USA) were used, respectively. To detect these signals on blotted membranes, the ECL Prime Western Blotting Detection System (GE Healthcare, Little Chalfont, UK) and LAS1000 (FUJIFILM, Tokyo, Japan) were used.

### Microscopy Experiments

For SEM analysis, strains were grown according to the same procedure as that in the calculation of conidia number. After 48 h of incubation at 37°C, appropriate-sized agar blocks were prefixed with 2% (w/v) glutaraldehyde containing 0.1 M phosphate buffer for 24 h, followed by 1% osmium tetraoxide in phosphate buffer for 1 h. Samples were dehydrated through a graded ethanol series to anhydrous 100% ethanol, substituted with isoamyl acetate, and then dried by the critical-point method (EM CPD030; Leica). After sputter coating with platinum (E102 Ion sputter, Hitachi, Tokyo, Japan), the samples were visualized under an S-3400 Scanning Electron Microscope (Hitachi).

For TEM analysis, hyphae that were cultured for 18 h were prefixed with 2% (w/v) glutaraldehyde containing 0.1 M phosphate buffer at 4°C for 10 h, followed by 1% osmium tetraoxide in phosphate buffer for 1 h. After dehydration, tissues were embedded in epoxy resin. Ultrathin sections were stained with uranyl acetate and lead citrate, and were visualized under a JEM 1400 electron microscope (JEOL, Tokyo, Japan). The hyphal diameter and cell wall thickness were measured using SMileView software (JEOL).

### Determination of Cell Wall Carbohydrate Composition

The cell wall fractionation by alkali treatment and quantitative determination of the carbohydrate composition of the fractions were performed as previously described [56]. Briefly, WT and Δ*nikA* strains were cultured in 1 L of YGMM for 24 h, harvested, squeezed, and freeze-dried. One gram of the mycelial powder was used for fractionation of cell walls by alkali treatment. All of the fractions (HW: hot-water soluble, AS1 and AS2: alkali-soluble, AI: alkali-insoluble) were freeze-dried, and the weight was measured. Each freeze-dried cell wall fraction was hydrolyzed by H_2_SO_4_ at 100°C and neutralized with barium carbonate. Finally, the carbohydrate composition of the samples was determined using a high-performance anion-exchange chromatography (HPAC) system.

## Supporting Information

Figure S1 **Radial growth rate on plates containing fungicides.** Growth inhibitory effects of fludioxonil (Flu), iprodione (Ipr), and pyrrolnitrin (PN) were examined. The WT strain was inoculated on YGMM agar with or without the indicated concentration of chemicals and grown at 37°C for 40 h. The radial growth rates were calculated by comparing the diameter of a colony to that of a colony grown on YGMM without chemicals. Each plot shows the mean based on three replicates.
(TIF)

Figure S2 **Growth of the** ***Co-nikA*** **strain under high osmotic or fungicide stress conditions.** Conidia of *Co-nikA* were inoculated onto YGMM containing sorbitol, NaCl, iprodione (Ipr), or fludioxonil (Flu) at the indicated concentrations and were incubated at 37°C for 44 h.
(TIF)

Figure S3 **Resistance to cell wall-perturbing reagents. (A)** Conidia of Δ*sakA*, Δ*sskA*, Δ*nikA*, and WT were inoculated onto YGMM containing 1 ng/mL micafungin (MCFG), 30 µg/mL congo red (CR), or 15 µg/mL calcofluor white (CFW) and incubated at 37°C for 48 h.
(TIF)

Figure S4 **Time-course expression of** ***catA*** **,** ***dprA*** **, and** ***dprB*** **in response to osmotic shock and fungicide. (A** and **B)** The WT strain was grown for 18 h at 37°C; then, sorbitol (A) or fludioxonil (B) was added (1 M and 10 µg/mL final concentrations, respectively). The mycelia were harvested at the indicated time points. The expression ratios were investigated by real-time RT-PCR. Relative expression ratios were calculated relative to the 0 min sample. Error bars represent the standard deviations based on three independent replicates.
(TIF)

Figure S5 **Comparison of growth of** ***A. fumigatus*** **HK gene deletion mutants under osmotic or fungicide stress conditions. (A)** Growth on plates containing hyperosmotic stress. Conidia of WT (Afs35), Δ*nikA*, Δ*fos1*, Δ*phkB*, Δ*fhk5*, and Δ*fhk6* were inoculated onto YGMM containing 1.2 M sorbitol and 1 M NaCl and incubated at 37°C for 72 h. Conidia of WT (Af293) and Δ*phkA* were inoculated onto YGMM containing 1.2 M sorbitol and were incubated at 37°C for 72 h. **(B)** Growth on plates containing fungicides. Conidia of WT (Afs35), Δ*nikA*, Δ*fos1*, Δ*phkB*, Δ*fhk5*, Δ*fhk6*, WT (Af293), and Δ*phkA* were inoculated onto YGMM containing 5 µg/mL iprodione (Ipr), 0.1 µg/mL fludioxonil (Flu), or 0.02 µg/mL pyrrolnitrin (PN), and were incubated at 37°C for 48 h.
(TIF)

Table S1 Radial growth of each strain on the plate containing fungicide or osmotic stress.
(XLSX)

Table S2 Cell wall composition of each cell wall fraction.
(XLSX)

Table S3 PCR primers used in this study.
(XLSX)
