# Supplementary figures and images for: NikA/TcsC Histidine Kinase Is Involved in Conidiation, Hyphal Morphology, and Responses to Osmotic Stress and Antifungal Chemicals in Aspergillus fumigatus
Source: PLoS One. 2013 Dec 2;8(12):e80881. doi: 10.1371/journal.pone.0080881 (PMC3846623; doi:10.1371/journal.pone.0080881)

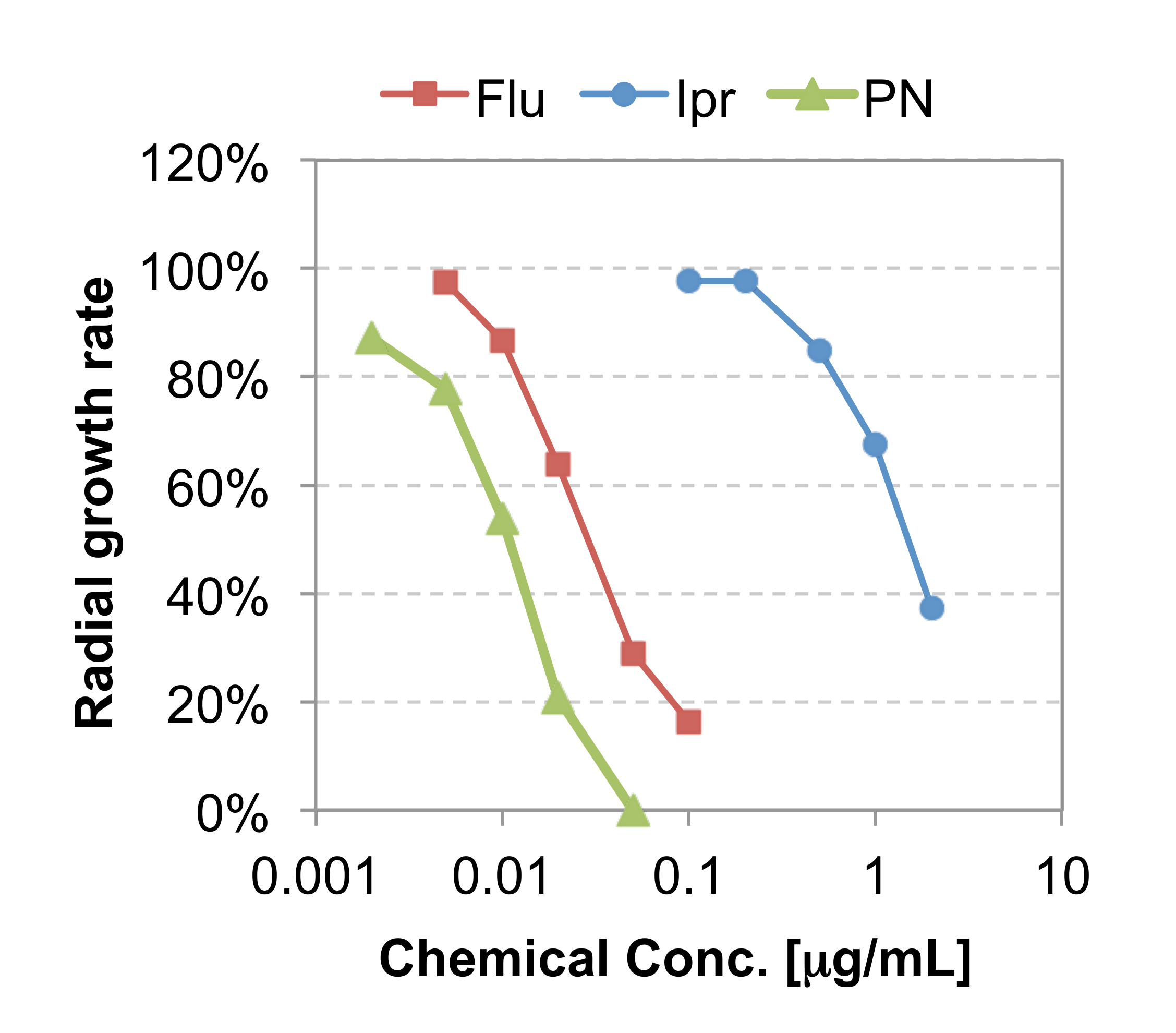

Supplement: Figure S1 — Radial growth rate on plates containing fungicides. Growth inhibitory effects of fludioxonil (Flu), iprodione (Ipr), and pyrrolnitrin (PN) were examined. The WT strain was inoculated on YGMM agar with or without the indicated concentration of chemicals and grown at 37°C for 40 h. The radial growth rates were calculated by comparing the diameter of a colony to that of a colony grown on YGMM without chemicals. Each plot shows the mean based on three replicates. (TIF) [file pone.0080881.s001.tif]

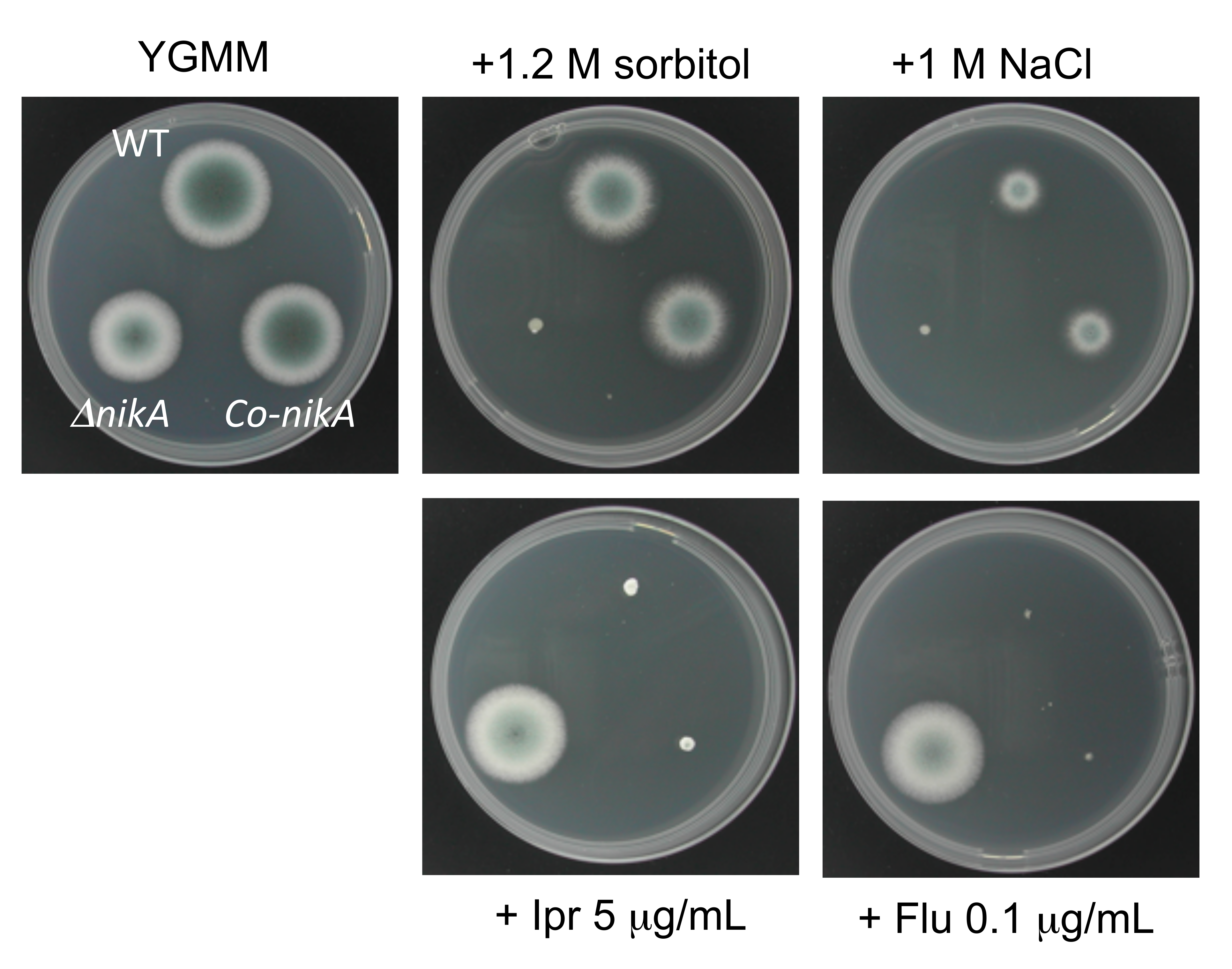

Supplement: Figure S2 — Growth of the Co-nikA strain under high osmotic or fungicide stress conditions. Conidia of Co-nikA were inoculated onto YGMM containing sorbitol, NaCl, iprodione (Ipr), or fludioxonil (Flu) at the indicated concentrations and were incubated at 37°C for 44 h. (TIF) [file pone.0080881.s002.tif]

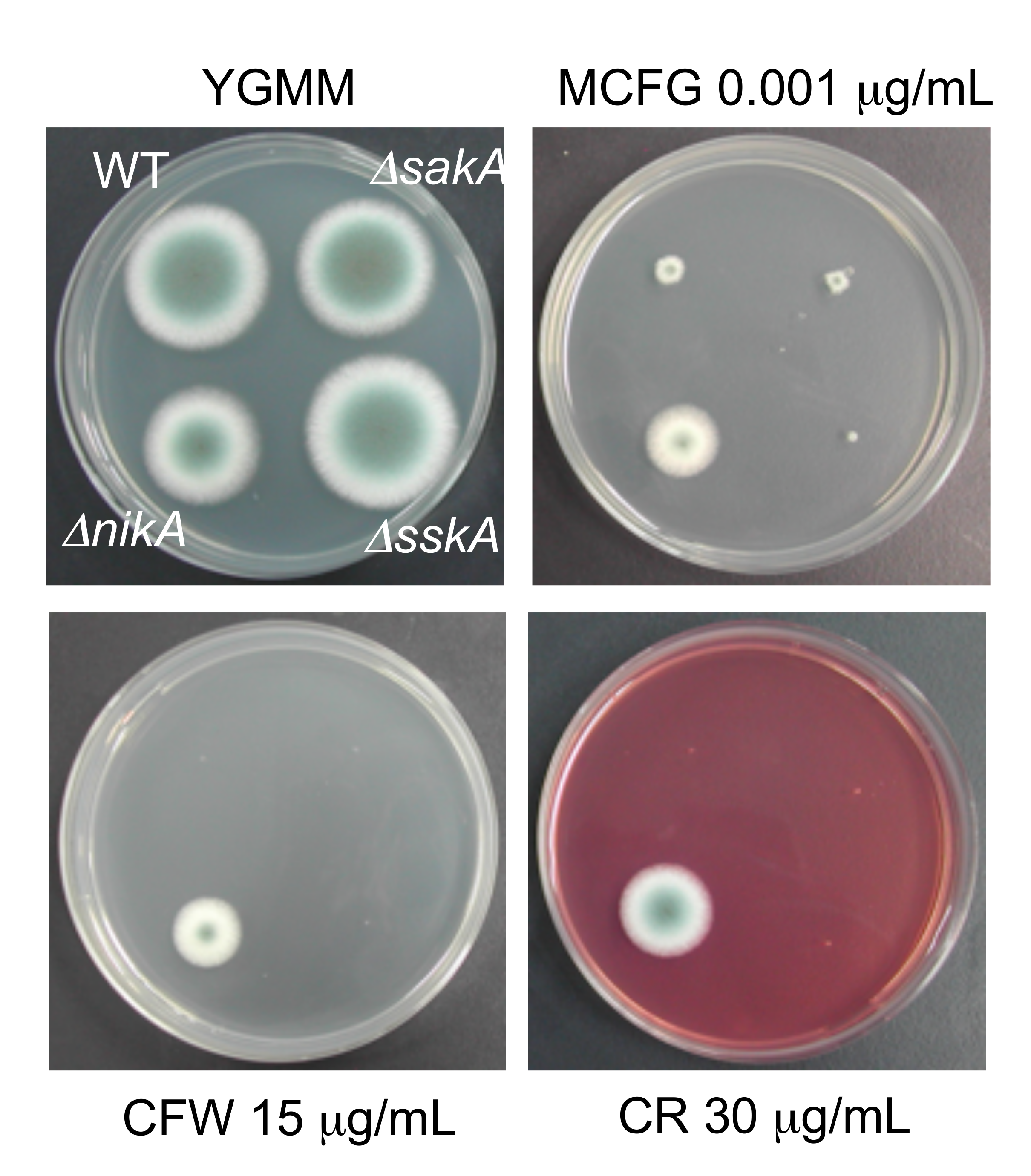

Supplement: Figure S3 — Resistance to cell wall-perturbing reagents. (A) Conidia of ΔsakA, ΔsskA, ΔnikA, and WT were inoculated onto YGMM containing 1 ng/mL micafungin (MCFG), 30 µg/mL congo red (CR), or 15 µg/mL calcofluor white (CFW) and incubated at 37°C for 48 h. (TIF) [file pone.0080881.s003.tif]

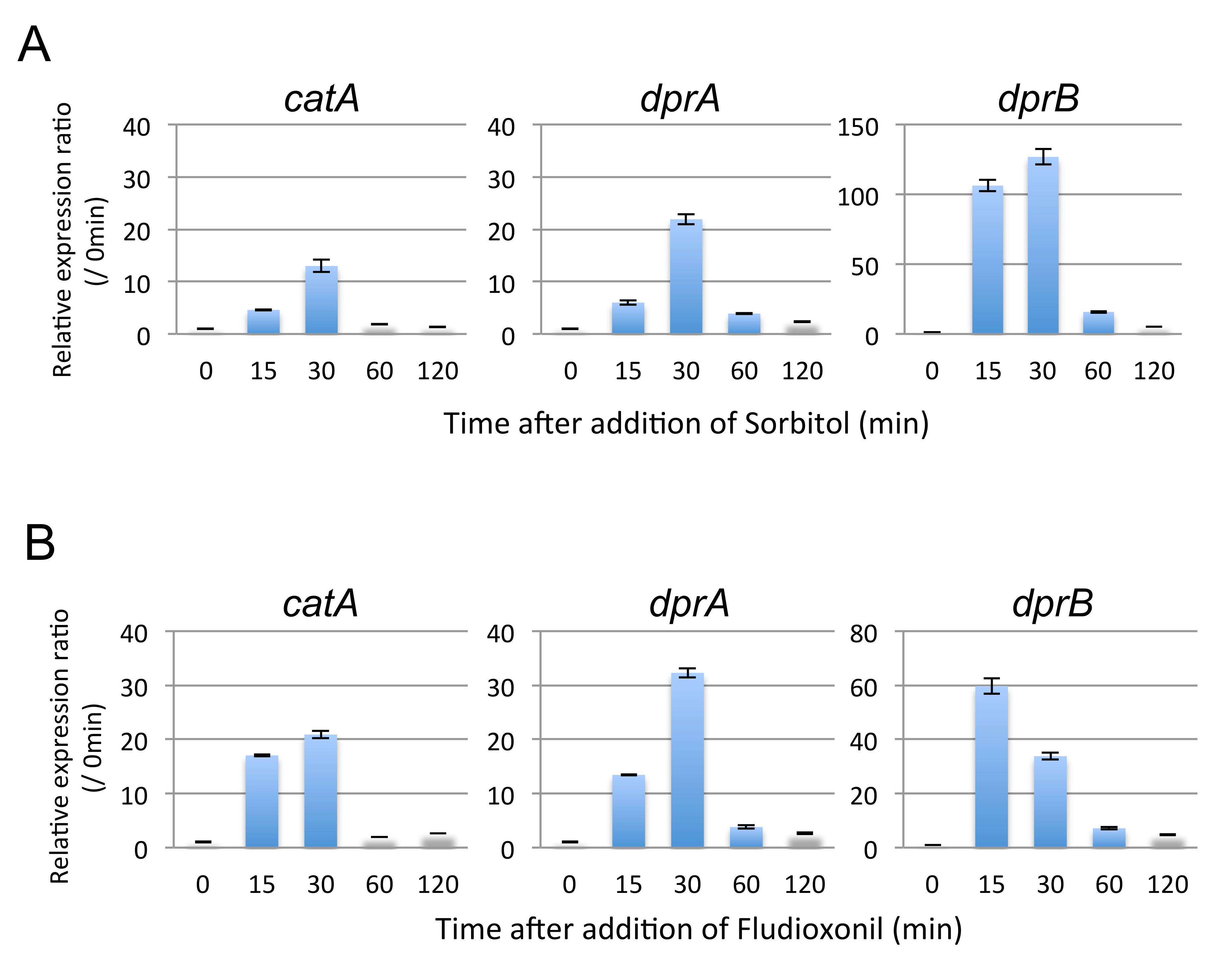

Supplement: Figure S4 — Time-course expression of catA , dprA , and dprB in response to osmotic shock and fungicide. (A and B) The WT strain was grown for 18 h at 37°C; then, sorbitol (A) or fludioxonil (B) was added (1 M and 10 µg/mL final concentrations, respectively). The mycelia were harvested at the indicated time points. The expression ratios were investigated by real-time RT-PCR. Relative expression ratios were calculated relative to the 0 min sample. Error bars represent the standard deviations based on three independent replicates. (TIF) [file pone.0080881.s004.tif]

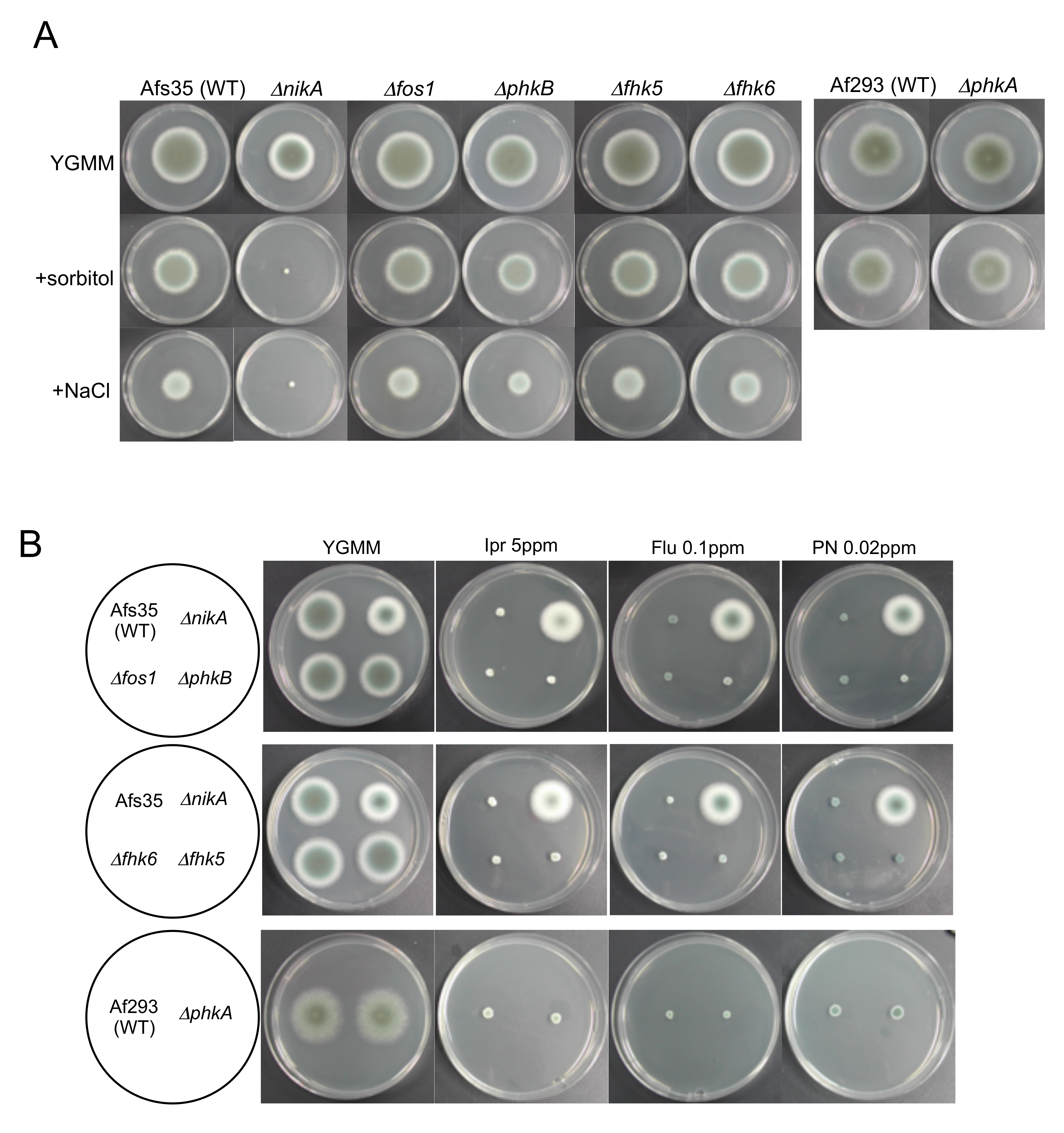

Supplement: Figure S5 — Comparison of growth of A. fumigatus HK gene deletion mutants under osmotic or fungicide stress conditions. (A) Growth on plates containing hyperosmotic stress. Conidia of WT (Afs35), ΔnikA, Δfos1, ΔphkB, Δfhk5, and Δfhk6 were inoculated onto YGMM containing 1.2 M sorbitol and 1 M NaCl and incubated at 37°C for 72 h. Conidia of WT (Af293) and ΔphkA were inoculated onto YGMM containing 1.2 M sorbitol and were incubated at 37°C for 72 h. (B) Growth on plates containing fungicides. Conidia of WT (Afs35), ΔnikA, Δfos1, ΔphkB, Δfhk5, Δfhk6, WT (Af293), and ΔphkA were inoculated onto YGMM containing 5 µg/mL iprodione (Ipr), 0.1 µg/mL fludioxonil (Flu), or 0.02 µg/mL pyrrolnitrin (PN), and were incubated at 37°C for 48 h. (TIF) [file pone.0080881.s005.tif]
